# The impact of sarcopenic obesity on knee and hip osteoarthritis: a scoping review

**DOI:** 10.1186/s12891-018-2175-7

**Published:** 2018-07-28

**Authors:** Kristine Godziuk, Carla M. Prado, Linda J. Woodhouse, Mary Forhan

**Affiliations:** 1 Faculty of Rehabilitation Medicine, University of Alberta, 8205 – 114 Street, 2-64 Corbett Hall, Edmonton, AB T6G 2G4 Canada; 2 Division of Human Nutrition, Faculty of Agricultural, Life and Environmental Sciences, University of Alberta, Edmonton, AB Canada; 3 Department of Physical Therapy, Faculty of Rehabilitation Medicine, University of Alberta, Edmonton, AB Canada; 4 Department of Occupational Therapy, Faculty of Rehabilitation Medicine, University of Alberta, Edmonton, AB Canada

**Keywords:** Sarcopenic obesity, Body composition, BMI, Osteoarthritis, Arthroplasty

## Abstract

**Background:**

The progressive, debilitating nature of knee and hip osteoarthritis can result in severe, persistent pain and disability, potentially leading to a need for total joint arthroplasty (TJA) in end-stage osteoarthritis. TJA in adults with obesity is associated with increased surgical risk and prolonged recovery, yet classifying obesity only using body mass index (BMI) precludes distinction of obesity phenotypes and their impact on surgical risk and recovery. The sarcopenic obesity phenotype, characterized by high adiposity and low skeletal muscle mass, is associated with higher infection rates, poorer function, and slower recovery after surgery in other clinical populations, but not thoroughly investigated in osteoarthritis. The rising prevalence and impact of this phenotype demands further attention in osteoarthritis treatment models of care, particularly as osteoarthritis-related pain, disability, and current treatment practices may inadvertently be influencing its development.

**Methods:**

A scoping review was used to examine the extent of evidence of sarcopenic obesity in adults with hip or knee osteoarthritis. Medline, CINAHL, Web of Science and EMBASE were systematically searched from inception to December 2017 with keywords and subject headings related to obesity, sarcopenia and osteoarthritis.

**Results:**

Eleven studies met inclusion criteria, with indications that muscle weakness, low skeletal muscle mass or sarcopenia are present alongside obesity in this population, potentially impacting therapeutic outcomes, and TJA surgical risk and recovery.

**Conclusions:**

Consideration of sarcopenic obesity should be included in osteoarthritis patient assessments.

## Background

Osteoarthritis is a chronic, progressive joint disease and leading cause of pain and mobility disability for over 27 million Americans [1] and 4 million Canadians [2]. Age, sex, genetics, joint trauma, and obesity all influence the development of this disease [3], and its progressive nature means advanced treatment options may be required in later stages to reduce pain, improve function and maintain quality of life. Surgical replacement of articular joint components, called a total joint arthroplasty (TJA), is currently the most effective treatment for severe pain and disability associated with end-stage knee or hip osteoarthritis that ceases to respond to other therapeutic interventions.

There has been a rapid and sustained increase in demand for TJA surgery around the world over the past two decades. TJA rates in the USA doubled from 336,000 patients in 1993 to 735,000 patients in 2005 [4], and are projected to top 4 million patients by 2030 [5]. In Canada, volumes are lower but the accrual rate tripled from 42,000 patients in 2000 [6] to 117,000 patients in 2016 [7], and similar persistent growth is apparent throughout Europe [8]. This increased demand is outpacing the supply of TJA, leading to longer wait times and pressure on health care systems to reduce delays in accessing care. To ensure timely and appropriate TJA access, optimization and prioritization of patient selection is critical. Clear, evidence-based guidelines for surgical appropriateness are lacking, resulting in a reliance on clinical judgement [9]. This has led to subjectivity in risk stratification, conflicting approaches and barriers or delays in treatment access for patients with obesity due to evidence of increased surgical risk.

Two meta-analyses have found increased risk of superficial infections (OR 1.7–2.2) [10, 11] and deep infections (OR 2.4) [10] after total knee arthroplasty (TKA) in patients with obesity (defined as a body mass index/BMI ≥ 30 kg/m^2^) compared to patients without obesity (BMI < 30 kg/m^2^). Those with severe obesity (BMI ≥ 40 kg/m^2^) appear to be at even higher risk, with four times the rate of infection after TKA compared to those without obesity [11, 12]. Increased infection after total hip arthroplasty (THA) is less clear [13]. Yet controversy exists around evidence of increased risk related to excess body weight. Methodological concerns regarding quality and comparability of studies have been raised, with underpowered sample sizes, BMI categorization/dichotomization, and absence of sub-classification by comorbidity status limitations in current evidence [14, 15].

Suggestions for establishing a BMI threshold for withholding TJA surgery have been made [11, 14, 16], while others argue against using BMI as an outright contraindication for TJA [17, 18]. Without clear guidelines, orthopaedic surgeons may decide to deny or delay surgery based on their interpretation of evidence of surgical risk. Of greater concern, many surgeons recommend that patients lose weight to reduce their BMI before returning for re-assessment of surgical eligibility [12, 14, 19]. This recommendation is in contrast to current evidence that suggests weight loss does not improve perioperative TJA risk. Lui et al. [20] found weight loss of ≥5% of body weight in the year prior to TJA resulted in either no difference or an increased risk of deep infection (OR 3.8). Weight loss may inadvertently increase perioperative infection, as muscle lost concomitantly with fat may lower lean muscle reserves, which are critical to the wound healing process [21].

Reliance on BMI may result in misclassification bias and denial of surgery for patients with obesity. BMI is a poor indicator of individual health as it cannot discern individual body composition of muscle, bone or fat [22]. Significant deviations in body composition within BMI categories have been reported [22–24], including twofold differences in adiposity [25] and 30 kg differences in lean soft tissue [26] between patients who have the same BMI [27]. Relying on BMI as a screening tool for TJA ignores the influence body composition has on surgical risk, particularly in relation to the amount of skeletal muscle mass as shown in other clinical scenarios [28, 29]. A high BMI could disguise important skeletal muscle mass depletion, as in the condition of sarcopenic obesity [26, 30].

### What is sarcopenic obesity?

Sarcopenic obesity is defined as the co-occurrence of high adiposity and sarcopenia, a term coined to describe low skeletal muscle mass, strength and physical function originally diagnosed in the elderly [31], but present across the age spectrum [32, 33]. Sarcopenia is associated with physical disability, falls, extended hospital stays, infection and non-infection related complications, and increased overall mortality [34–36]. Importantly, sarcopenia is not restricted to people who appear thin or underweight. Aging is often paralleled by increased rates of muscle loss and concomitant gains in adiposity (both subcutaneous and intramuscular), which can culminate in sarcopenic obesity [37].

Compounding the effects of both sarcopenia and obesity, sarcopenic obesity is associated with poorer quality of life and greater disability, morbidity and mortality when compared with either obesity or sarcopenia alone [37–39]. Although the majority of studies to date have been conducted in elderly individuals, sarcopenia and sarcopenic obesity are not limited to this population. There are several clinical disorders where individuals are prone to muscle loss (with or without concurrent obesity), including diabetes, cancer, chronic obstructive pulmonary disease, HIV, cirrhosis, and arthritis [40]. The presence of sarcopenic obesity may be particularly important to consider when surgery is indicated. In addition to increased length of hospital stay and increased mortality associated with this condition [40], there is convincing evidence of its relationship with increased infection rates [28, 29, 41].

With obesity present in 26 to 38% of adults in Canada and the USA respectively [42], and an aging population with a longer life span, sarcopenic obesity may be a new epidemiological trend of current times [43]. Importantly, it cannot be identified by simply measuring body weight or calculating BMI [44].

### Is sarcopenic obesity a concern in osteoarthritis?

**Fig. 1:**
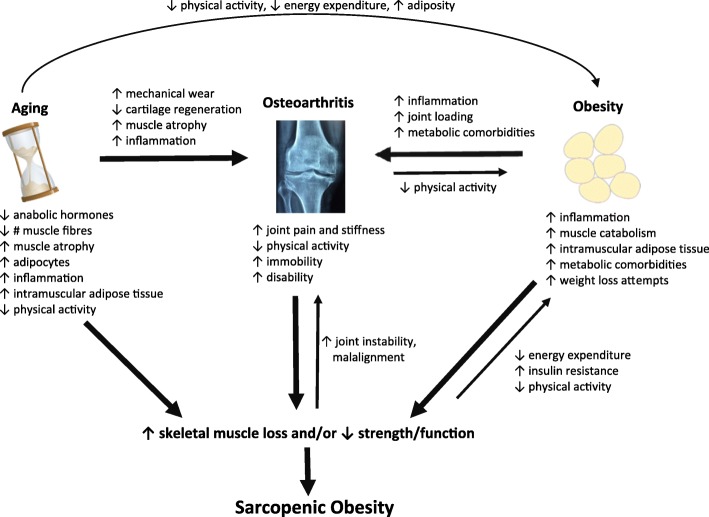
Relationship between aging, obesity and osteoarthritis and the development of sarcopenic obesity

Individuals with osteoarthritis may be at particular risk for sarcopenic obesity. The prevalence of osteoarthritis rises with age and obesity, and osteoarthritis-related pain can lead to inactivity and a decline in physical function. These factors in combination create a vicious cycle of inflammation, inactivity and aging-related muscle loss accompanied by aging-related gains in adiposity, giving rise and perpetuating the sarcopenic obesity phenotype [45–47] (Fig. 1). Chronic diseases associated with osteoarthritis [48], such as diabetes, metabolic syndrome, and hypertension, along with weight loss and subsequent re-gain (weight cycling), could exacerbate skeletal muscle loss, increase adiposity and contribute to the development of sarcopenic obesity [49]. Further, the development and progression of sarcopenia and osteoarthritis may occur through interrelated pathways [50, 51].

Body composition phenotypes of low skeletal muscle and high adiposity have been reported in patients with knee and hip osteoarthritis by Karlsson [52–54], Purcell [55] and Visser [56], although sarcopenia or obesity were not specifically identified. Nevertheless, this is compelling evidence and may indicate that this condition is present in osteoarthritis but not recognized or identified as sarcopenic obesity.

To provide a more complete understanding of sarcopenic obesity in lower extremity osteoarthritis, a scoping review was conducted to determine the extent of reported prevalence and impact of low muscle mass, muscle weakness or sarcopenia in adults with obesity and knee or hip osteoarthritis. Scoping reviews enable a comprehensive and encompassing review of emerging literature on a topic [57], and can be preferable to systematic reviews when the research question is examining the breadth of evidence on a topic, as in this case. Scoping reviews utilize transparent processes and systematic search strategies much like systematic reviews, and while they don’t typically include a grading system or formal quality assessment of included studies, a description of study limitations can be incorporated into the results.

## Methods

This scoping review was conducted following the methodology of Arksey and O’Malley [58], including a systematic search of the published literature. Medline, CINAHL, Web of Science and Embase databases were searched from inception to December 2017 using MeSH terms and keywords related to osteoarthritis, obesity, and sarcopenia (including dynapenia, muscle weakness, muscle atrophy, low muscle mass, muscle loss, body composition, body compartment, lean soft tissue, lean body mass, lean mass, fat free mass, muscle size or muscle mass). Inclusion criteria was determined by the authors prior to search initiation. Studies were to be included if they were primary or secondary analyses, and subjects had knee or hip osteoarthritis. Additionally, studies must have conducted group/subgroup analysis by obesity (identified using body mass index/BMI, waist circumference, fat mass or percent body fat), and examined muscle mass, muscle strength/weakness or sarcopenia. Studies on animal models and children were excluded, along with studies where participants did not have knee or hip osteoarthritis, or obesity, or if the study was an editorial, protocol or review article. Reference lists of relevant articles were hand searched to identify articles missed in the primary investigation. From each included study we extracted the author, publication year, study design, sample population, methodologies for assessing obesity and sarcopenia, study limitations and relevant findings. A summary of extracted information was tabulated and a descriptive analysis was conducted.

## Results

**Fig. 2:**
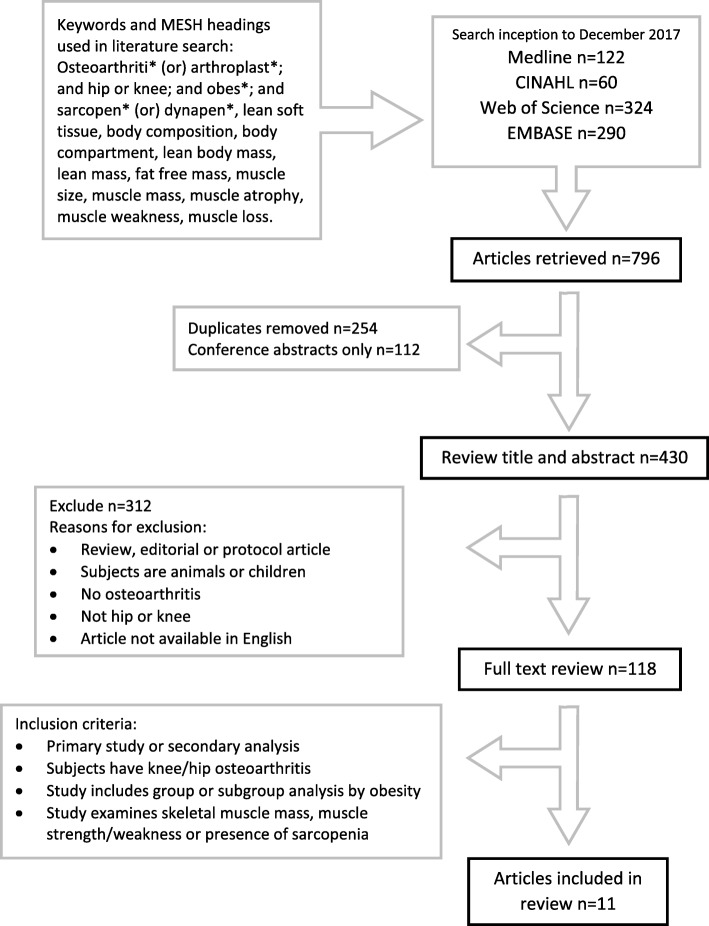
Systematic search strategy and results

A total of 796 articles were identified in the original search and 118 full text articles were screened for potential relevance (Fig. 2). Eleven studies met inclusion criteria [59–69], and a summary of study characteristics and key findings are presented in Table 1.

**Table 1:** Studies reporting low skeletal muscle mass and/or muscle weakness in adults with obesity and knee or hip osteoarthritis

Author, Year	Study purpose	Study design	Population	Definition of obesity	Body composition methodology	Definition of low muscle mass^a,b^ or muscle weakness	Study limitations	Relevant findings
Batsis et al. [59], 2015	To describe the impact of dynapenic obesity on physical function in knee OA	Longi-tudinal,	North American population from OsteoArthritis Initiative (OAI), age ≥ 60 years, *n* = 526 in subgroup with knee OA (rKOA),	BMI ≥30 kg/m^2^	NI	Lowest sex-specific tertile of knee extensor strength (dynapenia)	Secondary analysis of prospective data from longitudinal cohort. Excluded severe knee OA. No assessment of muscle mass or body composition	Prevalence of dynapenic obesity was 16%.
Clemence et al. [69], 2017	To analyze the association between low lean mass and clinical symptoms in knee and hip OA	Cross-sectional	French adults with hip and knee OA (KL grade ≥ 2) from KHOALA study, *n* = 358, age 63.4 ± 8.4 years	BMI ≥30 kg/m^2^, or sex specific FM or WC cut-offs	DXA	ASM/BMI < 0.789 for men and < 0.512 for women (FNIH cutoffs)	Secondary analysis of prospective data from longitudinal cohort. No information on exclusion criteria. No assessment of muscle strength or function	SO prevalence was 16.2%. Low lean mass was associated with pain and impaired function in subjects with normal BMI, but not with obesity (no significant differences between NSO and SO groups).
Ji et al. [60], 2016	To identify the prevalence of SO in knee and hip orthopedic surgery (OS) patients	Cross-sectional	Korean orthopedic surgery patients (hip or knee TJA or femoral fracture repair) (OS, *n* = 222) compared to control non-surgical outpatients (non-OS, *n* = 364)	BMI > 25 kg/m^2^	DXA	ASM/height^2^, ASM/weight, and ASM/height and fat mass (residuals)	Retrospective analysis of data. No assessment of muscle strength or function	SO prevalence ranged from 1.3–35.4% in TKA and 0–18.4% in THA patients depending on definition used. SO rates were higher in OS patients compared to non-OS patients.
Jin et al. [61], 2017	To examine the associations between obesity, sarcopenia and OA in elderly	Cross-sectional	Korean population (KNHANES) age ≥ 65 years group with knee OA (K/L grade ≥ 2) (*n* = 1865) compared to lumbar spondylosis group (*n* = 1709)	BMI ≥25 kg/m^2^	DXA	ASM/weight, 2SDs below average of sex-matched young reference group	Secondary analysis of population survey data. No assessment of muscle strength or function	Results indicate correlation between SO and NSO with knee OA, but no relationship with lumbar spondylosis. Females with SO had increased OR for knee OA when adjusted for age and waist circumference (OR 1.80, CI 1.03–3.12).
Knoop et al. [62], 2011	To identify distinct clinical phenotypes and their impact in knee OA	Cross-sectional	North American population with knee OA (K/L grade 0–4) from OsteoArthritis Initiative (*n* = 842, age 63.2 ± 9.1)	BMI ≥30 kg/m^2^	NI	Low mean score of quadriceps and hamstring isometric strength	Secondary analysis of prospective data from longitudinal cohort. No assessment of muscle mass or body composition. No clear cut-off for defining weakness	Dynapenic obesity group (“obese and weak” phenotype) had higher pain and poorer physical function compared to “minimal joint disease”, “strong muscle”, and “non-obese and weak” phenotypes.
Lee et al. [63], 2016	To investigate association between lower limb muscle mass and knee OA	Cross-sectional	Korean population (KNHANES) age ≥ 50 years, n = 821 with knee OA (K/L grade ≥ 2), (*n* = 821), and control group without knee OA (*n* = 4103)	BMI ≥27.5 kg/m^2^	DXA	ASM/weight, 2SD below the mean in sex-matched young reference group (< 29.5% in men, < 23.2% in women)	Secondary analysis of population survey data. No assessment of muscle strength or function	SO prevalence was 5.2% in knee OA group compared to 1.8% in control group.
Lee et al. [64], 2012	To analyze the association between knee OA, sarcopenia and obesity	Cross-sectional	Korean population (KNHANES) with bilateral knee OA (K/L grade ≥ 2) age ≥ 50 years, *n* = 2893	BMI ≥27.5 kg/m^2^	DXA	ASM/weight, 2SD below the mean in sex-matched young reference group (< 26.8% in men, < 21% in women)	Secondary analysis of population survey data. No assessment of muscle strength or function	SO prevalence was 3% overall. When adjusted for age and sex, SO had stronger association with knee OA (OR 3.51, CI 2.15–5.75) compared to NSO (OR 2.38, CI 1.80–3.15).
Manoy et al. [65], 2017	To assess association between leptin, vitamin D, muscle strength and physical performance in knee OA	Cross-sectional	Thailand knee OA patients (K/L grade < 3) (*n* = 208), age 65 ± 7 years	BMI > 25 kg/m^2^	BIA	ASM/weight < 30.4% in men and < 25.8% in women, and EWGSOP gait speed and grip strength cutoffs	Unclear if data collected retrospectively or prospectively. No description of sampling methods. Excluded severe knee OA	SO prevalence was 13.9%. Patients with SO had poorer performance on the timed up and go (TUG), sit to stand (STS) and 6 min walk tests (6MWT) compared to those with NSO or NO.
Oosting et al. [66], 2016	To determine the association of obesity and recovery after THA when stratified by muscle strength	Cross-sectional	Netherlands THA patients (*n* = 297), age 69 ± 11 years	BMI > 30 kg/m^2^	NI	Maximal handgrip strength (< 20 kg for woman and < 30 kg for men)	Secondary analysis of prospective cohort. No assessment of muscle mass or body composition	Obesity and muscle weakness (dynapenic obesity) was associated with prolonged length of stay > 4 days (OR 3.59, CI 1.09–11.89) and delayed inpatient recovery (> 2 days to walk with gait aid) (OR 6.21, CI 1.64–23.65), but not in those with obesity alone.
Segal et al. [67], 2005	To analyze the impact of low limb lean mass in knee OA distinct from body weight	Cross-sectional	Japanese female orthopedic knee OA (K/L grade ≥ 2) patients age ≥ 45 years (*n* = 341), compared to control group with fracture, sprains or back pain (*n* = 604)	BMI > 24.9 kg/m^2^	BIA	Lower limb LST	Unclear if data collected retrospectively or prospectively. No clear cut-off for defining low LST. No assessment of muscle strength or function	Females with knee OA had 5–15% less lower limb LST compared to control groups across BMI categories, with significant 1.8 kg and 1.5 kg differences in overweight and obesity groups, respectively.
Suh et al. [68], 2016	To analyze the association between obesity, sex, and lower extremity lean mass in knee OA	Cross-sectional	Korean population (KNHANES) age ≥ 50 years with unilateral knee OA (K/L grade ≥ 2) (*n* = 4246; 1829 men and 2417 women)	BMI ≥27.5 kg/m^2^	DXA	Lower extremity LST/weight, in lowest quartile	Secondary analysis of population survey data. No assessment of muscle strength or function	In females, obesity and low muscle mass was strongly association with knee OA (OR 2.31, CI 1.35–3.93) compared to obesity and normal muscle mass (OR 1.03, CI 0.26–4.02).

*ASM* appendicular skeletal mass, *ASMI* ASM/height^2^, *BIA* bioelectrical impedance analysis, *BMI* body mass index, *CI* confidence interval, *DXA* dual-energy x-ray absorptiometry, *EWGSOP* European Working Group on Sarcopenia in Older People, *FM* fat mass, *FFM* fat free mass, *FNIH* Foundation for the National Institute of Health, *KNHANES* Korean National Health and Nutrition Examination Survey, *K/L* Kellgren/Lawrence radiographic osteoarthritis score, *LST* lean soft tissue, *LSTI* LST/height^2^, *NI* not included in study design, *NO* normal body composition, *NSO* non-sarcopenic obesity, *OA* osteoarthritis, *OR* odds ratio, *rKOA* radiographic evidence of knee osteoarthritis, *SD* standard deviation, *SO* sarcopenic obesity, *THA* total hip arthroplasty, *TJA* total joint arthroplasty, *TKA* total knee arthroplasty, *VAS* visual analog scale, *WC* waist circumference, *WOMAC* Western Ontario and McMaster Universities Osteoarthritis Index

^a^Varied indices for identifying low muscle mass: LSTI, LST/weight, ASM, ASMI, ASM/weight, ASM/BMI, ASM relative to height and FM (residuals), and FM:FFM ratio [26]. Indices that consider LST or ASM relative to weight, BMI or FM may be most appropriate in adults with obesity [26], and relevant to identify clinically relevant weakness [76]

^b^Terms from included studies were adjusted for consistency and accurate representation of body composition compartment, and may differ from original reports

Publication dates ranged from 2005 to 2017, with the majority (*n* = 8, 73%) published in the last three years, potentially indicating a growing awareness and understanding of sarcopenic obesity. Ten of the eleven studies were cross-sectional [60–69], and one longitudinal [59]. Four studies (36.4%) were secondary analyses of the Korea National Health and Nutrition Examination Survey (KNHANES) population cohort [61, 63, 64, 68], two (18.2%) were secondary analyses of the North American Osteoarthritis Initiative (OAI) population cohort [59, 62], one (9%) was a secondary analysis of the French Knee and Hip OsteoArthritis Long-term Assessment (KHOALA) cohort [69], and the remaining four (36.4%) were independent studies with cohorts from Korea [60], Thailand [65], Japan [67] and the Netherlands [66]. Eight studies focused on osteoarthritis of the knee joint [59, 61–65, 67, 68], with two additional studies examining both knee and hip [60, 69], and one solely on hip osteoarthritis [66].

## Discussion

This scoping review identified eleven studies with clear indications that muscle weakness, low skeletal muscle mass, or sarcopenia occur in conjunction with obesity in lower extremity osteoarthritis. The majority of included studies examined prevalence and association of the sarcopenic obesity phenotype with the presence of knee or hip osteoarthritis [60, 61, 63, 64, 67, 68], however others investigated the impact on pain, physical function, and quality of life [59, 62, 65, 69] or arthroplasty outcomes [66].

The prevalence of the sarcopenic obesity phenotype in adults with knee osteoarthritis may be as high as 35.4% [60], although a wide range was reported across included studies (prevalence of 3% [64], 13.9% [65], 16.2% [69], and up to 35.4% [60]). Differences in prevalence are likely related to varied obesity and sarcopenia classification criteria utilized in each study, a problem previously addressed elsewhere [26]. Obesity was classified by BMI (in kg/m^2^) in all studies, but different cut-offs were used in Asian populations (either BMI ≥ 25 [60, 61, 65, 67] or ≥ 27.5 [63, 68]), and North American and European populations (BMI ≥ 30 [59, 62, 66, 69]), making it difficult to compare across study groups and populations. Prevalence also varied depending on the sarcopenia assessment method used in the study. Ji et al. [60] examined differences in sarcopenic obesity rates in hip and knee arthroplasty patients comparing low muscle mass (assessed with dual-energy x-ray absorptiometry/DXA) using three approaches: appendicular skeletal mass (ASM)/height^2^, ASM/weight, and ASM relative to height and total fat mass, called the residual method [70]). They found prevalence of sarcopenic obesity differed between 1.3–35.4% in TKA patients and 0–18.4% in THA patients depending on the approach. Whether distinctions exist between low muscle mass present only in the lower extremities versus the whole body remains unclear [63, 67, 68]. Emerging evidence suggests that in patients with a larger body mass, the ratio between fat and muscle compartments (a metabolic load-capacity model) may be most relevant for identifying clinically important sarcopenic obesity [26].

There is currently no definitive diagnostic criteria established to identify sarcopenic obesity [71–73]. Several consensus papers on defining sarcopenia in the elderly have been published, including the European Working Group on Sarcopenia in Older Persons (EWGSOP) [31], the European Society for Clinical Nutrition and Metabolism Special Interest Groups (ESPEN-SIG) [74], the International Working Group on Sarcopenia (IWGS) [75], and the Foundation for the National Institute of Health (FNIH) [76]. There is general agreement that the presence or absence of sarcopenia in the elderly should be based on a combined assessment of physical function (measurement of gait speed), muscular strength (measurement of handgrip or lower body strength), and body composition (to determine low skeletal muscle mass). However whether these measures are equally applicable to patients with concurrent chronic degenerative conditions remains to be explored.

Of the studies in this scoping review, seven used only body composition/low muscle mass for sarcopenia identification [60, 61, 63, 64, 67–69], three used only an assessment of muscle weakness (testing handgrip [66] or quadriceps strength [59, 62]), and only one study utilized a combined approach following EWGSOP consensus criteria [65] including assessment of physical function with gait speed in addition to muscle strength and body composition. Using gait speed as an assessment of physical function may create challenges in the osteoarthritis population. Osteoarthritis-related joint pain and stiffness may impact testing methods or may require alterations or alternatives to currently used criteria thresholds [77] or modifications to gait speed parameters. Additionally, risk of falls is high in those with moderate to severe osteoarthritis [78], which may increase the challenge of assessing physical function in this population.

The relationship between the sarcopenic obesity phenotype and knee osteoarthritis may be unique compared to other orthopedic and musculoskeletal conditions. In the included studies, no association was found between sarcopenic obesity and lumbar spondylosis [61], or in patients with fractures, sprains and back pain [67], or non-orthopedic hospital outpatients [60]. The development and progression of sarcopenic obesity may be interrelated with osteoarthritis development and progression. Lee et al. [63] found sarcopenic obesity was more prevalent in Korean adults with knee osteoarthritis compared to those without knee osteoarthritis (5.2% vs 1.8%, respectively). Batsis et al. [59] found rates of muscle weakness with obesity were higher in adults with clinically diagnosed knee osteoarthritis compared to those at risk for knee osteoarthritis (16% vs 6%, respectively). Sex specific differences may exist in this relationship. Suh et al. [68] found increased odds of knee osteoarthritis when low lower-extremity muscle mass was present in women with obesity (OR 2.31, CI 1.35–3.93), but not in men. Another study reported similar associations only in women over age 65 [61].

The findings of this scoping review support the theoretical impact of sarcopenic obesity on therapeutic outcomes for osteoarthritis, and surgical risk and recovery after joint arthroplasty. To date, only one study has investigated outcomes after TJA, with results showing obesity with muscle weakness was related to delayed independent walking (more than 2 days) and prolonged hospital stays (more than 4 days) compared to obesity alone [66].

It is reasonable to infer that reduced muscle strength or skeletal muscle mass would influence short and long-term recovery after arthroplasty and rehabilitation requirements to return to daily life. Muscle depletion is indicative of a reduction in physiologic protein reserves, which can contribute to impaired wound healing, increased risk of infections and longer recuperation after surgery [79]. A study by Kumar et al. [80] found that handgrip strength < 15 kg was associated with longer hospital stay after TJA, highlighting this potential relationship. Further, a study by Mau-Moller et al. [81] reported that low thigh muscle mass was a better predictor than BMI for loss of bone mineral density after TKA. This is important as loss of bone mineral density can lead to early prosthetic loosening after TKA and a need for revision surgery, suggesting that muscle mass may be more relevant than BMI for long term TKA outcomes.

Identifying sarcopenic obesity early in the continuum of care for osteoarthritis is critical to avoid inappropriate treatment recommendations. The current practice of recommending weight loss prior to TJA based on assessment of body weight or BMI [64] may need further consideration as weight loss attempts may also result in loss of skeletal muscle mass [40, 49], potentially exacerbating the sarcopenic obesity phenotype. Body composition measurement may be a critical assessment tool to distinguish between normal versus abnormal amounts of skeletal muscle mass and provide a more accurate assessment of adiposity [82], as anthropometric measures of obesity (using waist circumference, height, weight and BMI) may not differentiate between muscle and adipose tissue compartments. As previously discussed, body weight loss ≥5% in year preceding TJA was associated with increased surgical risk and higher readmission rates [20]. This may be a result of individuals with sarcopenic obesity losing weight, further reducing their already low muscle reserve, in turn impacting healing rates and perpetuating the vicious cycle of sarcopenia and obesity. Alternatively, it could suggest individuals with obesity and normal skeletal muscle mass (non-sarcopenic obesity) became sarcopenic post weight-loss (by losing more skeletal muscle mass without a substantial decrease in body weight to be considered non-obese) [40].

### Study limitations

Every effort was made to comprehensively search and include all relevant studies in the literature, however there is a possibility that some were inadvertently missed. Further, while a limitation of scoping reviews is the lack of a formal risk of bias or study quality assessment, we have included a descriptive analysis of study design and limitations in Table 1 of the results section to enable assessment of level of evidence.

## Conclusion

Sarcopenic obesity may be impacting therapeutic and surgical outcomes in osteoarthritis treatment approaches, yet this cannot be discerned until assessments for sarcopenic obesity are explored and regularly applied. There is a need to move beyond BMI and simple obesity diagnosis in osteoarthritis models of care, possibly including more sophisticated assessments of body composition. As gait speed and handgrip strength assessments to identify patients at risk for sarcopenic obesity have not been well-tested in the osteoarthritis population, further research is required to clarify the effectiveness of these screening approaches in populations with physical function limitations. In the interim, incorporating clinical assessments for sarcopenic obesity through body composition may be essential to prevent misclassification bias and provide clarity on TJA surgical risk and recovery in adults with obesity.

## Abbreviations

BMI: body mass index
CINAHL: Cumulative Index of Nursing and Allied Health Literature
DXA: dual-energy x-ray absorptiometry
EMBASE: Excerpta Medica dataBASE
MeSH: medical subject heading
THA: total hip arthroplasty
TJA: total joint arthroplasty
TKA: total knee arthroplasty
